# Sodæ Sulphis in the Treatment of Intermittent Fever

**Published:** 1869-01

**Authors:** A. C. Simonton

**Affiliations:** Mitchellville, Iowa


ARTICLE II.

### SODÆ SULPHIS IN THE TREATMENT OF INTERMITTENT FEVER.

By A. C. SIMONTON, Mitchellville, Iowa, December 3, 1868.

In a communication to the July number of the EXAMINER, I gave the results of a short experience in the treatment of *chronic ague* with sulphite of soda; and, in the closing remarks, made a statement concerning its efficacy in the acute form of the disease. But since that time I have pushed these experiments still farther, and am now better prepared to give an opinion concerning the value of the remedy in this latter form of the disease.

I have not only made use of it, as stated in the former article, in connection with the ordinary antiperiodics, to render the cure more certain and permanent, but have thrown aside all other remedies in a great majority of cases treated during the autumn months; and I must say, the results have entirely surpassed my expectation.

My field for observation has been quite extensive, and the experiments have been confined to no small number of patients; therefore, I feel justified in laying the results before the profession.

Intermittent fever is a disease so peculiar in itself, that there is scarcely any possibility of being deceived concerning the value of a remedy in its treatment.

It does not belong to that class of, so-called, self-limited diseases; but, on the contrary, we are all aware of its tendency to grow from bad to worse, and continue indefinitely, especially if the patient continues to be exposed in a miasmatic district.

This being the fact, we are justifiable in classing any remedy as among the list of curative agents, which, when administered alone, cuts short the disease and restores patients to health, while yet exposed to those influences which naturally tend to produce and prolong the malady; and especially are we safe in coming to a conclusion of this kind, when the remedy has been tested in a great number of cases.

That the sulphite of soda is a remedy of this class, can no longer be doubted; and that it does the work as efficiently, and about as speedily, as the sulphate of quinia, is no longer to be denied.

The first case in which I ventured to administer the drug, alone, occurred August 13, 1868, when I was called to see a patient who had just emerged from a paroxysm of intermittent, and who was living in a strongly malarious section of the country. I determined in this case to try the efficacy of the sulphite, even at the risk of professional reputation. I therefore ordered for him 10-gr. doses of the sulphite of soda in solution, to be taken every two hours during the daytime, and every three or four hours during the night, right along, without regard to exacerbations or intermissions. The patient followed my directions strictly, and, to my great satisfaction, there was no recurrence of the paroxysms whatever.

I had him continue the remedy in 10-gr. doses, three times a day, for about eight days longer, in order to render the cure more permanent. And I will state right here, that this patient has not had another paroxysm of ague during all the autumn months, and up to this time of writing, December 3d; and furthermore, that according to his history, he has been quite susceptible to the influence of malaria during former seasons.

Being encouraged by the results in the case just mentioned, I prescribed it in another case, August 17, 1868. This patient had been laboring under the quotidian form of the disease for about a week, and, dreading to take the old remedy, quinine, he had neglected calling aid sooner. Taking advantage of his disgust for quinine, I prescribed the sulphite in 10-gr. doses in solution as before, and to be given in like manner. The patient persisted in its use, and only succeeded in arresting the disease after the third paroxysm from commencement of medicine.

In this case, the prescription was made just prior to the occurrence of a paroxysm, and of course we would expect no amelioration of symptoms during this exacerbation; but the next paroxysm came on later, and was of shorter duration; the third was still lighter, which terminated the disease.

Now it may be alleged, that if the remedy acted as efficiently, it did not act as promptly, as quinine would have done in this case; but this allegation has but little weight, when we recollect that in cases which have continued for a number of days, quinine does not abridge the disease at once, but frequently requires as much time to produce a cure as the sulphite did in this instance. I will state here also, as in former case, that there has been no return of ague up to this time.

On August 18th, the next day after I had prescribed for the last-mentioned case, I was called to see a young lady for whom I had ordered quinine for a simple intermittent a few days before. She had taken two or three doses of the quinine, but having a natural disgust for the remedy, declared she would not take any more. I therefore prepared for her the sulphite as in former cases, and she took it accordingly. The result was, that there was no recurrence of the paroxysms after commencing the remedy, save the one that was approaching at the time of prescribing. In this case, like the former, the remedy was continued in 10-gr. doses three times a day for several days, in order to completely eradicate the poison from the system. The patient remarked in a few days, that she felt better than she had all summer. There was no return of the disease during the autumn months.

I might go on and occupy several pages in reporting cases which I have treated with the sulphite, but it is hardly necessary; and there would be too much sameness about it, to be at all interesting, save as a mere matter of statistics. I have reported the three cases above as illustrative of my earliest experience with the remedy, alone, in the treatment of intermittents; and they will serve, also, to illustrate what may be expected from the remedy by those who have not ventured yet to give it a trial, and may wish to do so.

Since these cases were treated, it has been fully as common, during the autumn months, for me to prescribe the sulphite in simple intermittents, as it has quinine; and, in fact, I have almost felt as though I was doing my patients an injustice if I neglected to follow up the quinine treatment with sulphite.

During September and October, the cases treated by the sulphite, alone, were very numerous; and I feel confident in saying, that it has not failed in one single instance. And furthermore, I am fully persuaded that the remedy produces a more complete cure than is generally the case with quinine. This is certainly exemplified by the fact that relapses very seldom occur, at least in any short space of time, even though the patients are constantly exposed to the malarial influence.

As a matter of course, it is useless to assert that any remedy will render the system insusceptible to the poison, whatever its nature may be, which produces the disease under consideration; but when I assert that I have never observed but one case of relapse, out of the number treated by the remedy since

August last, I assert that which cannot be done concerning the number of cases treated by quinine, under the same circumstances. And in order that I might not be deceived on this point, I prescribed, on more than one occasion, quinine for one patient, and the sulphite for another, both being members of the same family, and, as a matter of course in such cases, surrounded by the same circumstances; and, as intimated above, relapses would occur in the cases treated with the quinine, when nothing of the kind would take place in those treated with the sulphite.

As regards the effects of the remedy on the various secretions, I have observed nothing of importance. The urine does not seem to be increased in quantity, although it is asserted by one or two observers, that some of its constituents are relatively altered in quantity. The effect on the bowels is very slight; the evacuations being rendered a little more free and copious, but, generally speaking, are not increased in frequency. The hyposulphite of soda, however, has a great tendency to cause looseness of the bowels, and for that reason I generally prefer the proto-salt. This fact, of the different effects of the two salts on the bowels, may be of some advantage occasionally, when this class of remedies is called into requisition.

It will be observed that I have said nothing about the remedy in the treatment of remittent fever; and the reason for this is, that my opportunities for observing its effects in this latter form of disease have been so limited, that I would not be justified in making any statement concerning this point; but I cannot see why the remedy would not be as admissible in remittent, as in the intermittent form of disease, since it is generally conceded by the profession that the two forms of disease are produced by identically the same poison.

Hoping to hear from other members of the profession on this important subject, I submit this meagre report for their consideration.

